# Gut pathobiont enrichment observed in a population predisposed to dementia, type 2 diabetics of Mexican descent living in South Texas

**DOI:** 10.3389/frmbi.2024.1456642

**Published:** 2024-12-03

**Authors:** Lisa M. Matz, Nisarg S. Shah, Laura Porterfield, Olivia M. Stuyck, Michael D. Jochum, Rakez Kayed, Giulio Taglialatela, Randall J. Urban, Shelly A. Buffington

**Affiliations:** ^1^ Center for Precision Environmental Health, Baylor College of Medicine, Houston, TX, United States; ^2^ Department of Internal Medicine, The University of Texas Medical Branch at Galveston, Galveston, TX, United States; ^3^ Department of Family Medicine, The University of Texas Medical Branch at Galveston, Galveston, TX, United States; ^4^ Sealy Institute for Vaccine Sciences, The University of Texas Medical Branch at Galveston, Galveston, TX, United States; ^5^ Department of Obstetrics and Gynecology, Baylor College of Medicine, Houston, TX, United States; ^6^ Department of Neurology, Mitchell Center for Neurodegenerative Diseases, The University of Texas Medical Branch at Galveston, Galveston, TX, United States; ^7^ Department of Neuroscience, Baylor College of Medicine, Houston, TX, United States

**Keywords:** type 2 diabetes, dementia, neurodegeneration, Alzheimer’s disease, comorbidity, Mexican American, microbiome, western diet

## Abstract

Type 2 diabetes (T2D) is a common forerunner of neurodegeneration and accompanying dementia, including Alzheimer’s Disease (AD), yet the mechanisms underlying this comorbidity remain unresolved. Individuals of Mexican descent living in South Texas have increased prevalence of comorbid T2D and early onset AD, despite low incidence of the APOE-ε4 risk variant among the population and an absence of a similar predisposition among relatives residing in Mexico – suggesting a role for environmental factors in coincident T2D and AD susceptibility. We therefore sought to test if differences in gut community structure could be observed in this population prior to any AD diagnosis. Here, in a small clinical trial (ClinicalTrials.gov Identifier NCT04602650), we report evidence for altered gut microbial ecology among subjects of Mexican descent living in South Texas with T2D (sT2D) compared to healthy controls without T2D (HC), despite no differences in expressed dietary preferences. We performed metataxonomic 16S rRNA gene amplicon sequencing of study participant stool samples. Although no significant decrease in microbial alpha diversity was observed between sT2D gut communities *versus* those of HC, body mass index was identified as a driver of gut community structure. Intriguingly, we observed a significant negative association of *Faecalibacterium* and *Lachnospiraceae* with T2D and an increase in the abundance of pathobionts *Escherichia-Shigella*, *Enterobacter*, and the erysipelotrichial species *Clostridia innocuum* among sT2D gut microbiota, as well as differentially abundant gene and metabolic pathways. Future large-scale, longitudinal sequencing efforts of the gut microbiome of individuals with T2D who go on to develop AD might identify key actors among “disease state” microbiota that contribute to increased susceptibility to comorbid dementia. Finally, we identified candidate microbiome-targeted approaches for the treatment of T2D.

## Introduction

Epidemiological studies identify type 2 diabetes (T2D) as a common antecedent of pathological neurodegeneration and accompanying dementia, including Alzheimer’s Disease (AD) (Biessels and Despa, 2018; Biessels and Whitmer, 2020; Srikanth et al., 2020); however, the mechanisms by which T2D increases risk for neurodegenerative disorders remains unknown. We performed a small clinical trial in individuals of Mexican descent living in South Texas. Although it is well established that this population is at increased risk of developing both T2D and, subsequently, comorbid AD compared to non-Hispanic whites (O'Bryant et al., 2013a; Johnson et al., 2015), the contributing factors and underlying pathophysiology remain unknown. The leading risk factor for T2D is obesity (Lillioja et al., 1993; Franks and McCarthy, 2016). According to the Centers for Disease Control (Center for Disease Control and Prevention, 2018), Texas ranks 14^th^ in the nation for obesity prevalence (Flegal et al., 2016). Within the Texan population, individuals of Mexican descent are disproportionately afflicted by obesity and T2D, with twice the prevalence of T2D (15.7%) relative to non-Hispanic whites (Umpierrez et al., 2007; Fisher-Hoch et al., 2010; Ross et al., 2015). Although a combination of genetic and environmental factors contribute to the etiology and pathophysiology of obesity (Franks and McCarthy, 2016) and comorbid T2D (Franks and McCarthy, 2016; Mambiya et al., 2019; Guzman-Castaneda et al., 2020), a recent study of 132 twin pairs found that – independent of genetics – overnutrition is the main factor underlying higher body mass index (BMI) (Berntzen et al., 2019). Furthermore, despite similar genetics, the Health and Aging Brain among Latino Elders (HABLE) study found the prevalence of abdominal obesity and T2D in Americans of Mexican descent to be significantly higher than in similarly aged patients in a strictly Mexican cohort in the Mexican Health and Aging Study (MHAS) (Vintimilla et al., 2020). This finding suggests that environmental factors associated with migration have a significant effect on metabolic health.

Mexican immigrants to the US and their descendants are faced with significant dietary changes, including exposure to a Western diet (Santiago-Torres et al., 2016). Characterized by increased animal protein and sugar consumption with decreased complex carbohydrate consumption, Western diet contributes to inflammation and pathological weight gain in diet-induced obesity (Mozaffarian et al., 2011; Longoria et al., 2022). Importantly, host diet-derived macronutrient availability regulates the composition of the human gut microbiome (David et al., 2014; Turnbaugh, 2017). The gut microbiome is emerging as a powerful regulator of host physiology (Clemente et al., 2012), including metabolic function (Nicholson et al., 2012; Tremaroli and Backhed, 2012; Vallianou et al., 2019), brain function, and behavior (Sampson and Mazmanian, 2015; Vuong et al., 2017; Jasarevic and Bale, 2019; Sherwin et al., 2019). Importantly, host diet-derived macronutrient availability regulates the composition of the human gut microbiome (David et al., 2014; Turnbaugh, 2017), and an anti-inflammatory diet was recently shown to reduce risk of dementia in patients with cardiometabolic disease by 31 percent (Dove et al., 2024). Although recent studies identify key gut microbiome signatures in patients with obesity and T2D (Turnbaugh et al., 2006; Turnbaugh et al., 2009), we sought to characterize the gut microbiome of individuals of Mexican descent living in the US, a population at increased risk of developing comorbid T2D and AD.

The potential contribution of nongenetic factors, such as changes in the functional composition of the gut microbiome, to AD prevalence in individuals of Mexican descent with T2D is supported by several recent studies. First, a meta-analysis of two large-scale studies comparing risk factors for mild cognitive impairment (MCI) in non-Hispanic Americans *versus* Mexican Americans found that among age, education, Apolipoprotein E (APOE) ε4 status, and gender, only advanced age was a significant risk factor for MCI in Mexican Americans (O'Bryant et al., 2013b). In a second study, analysis of serum samples identified divergent biomarker profiles in Mexican Americans with AD compared to non-Hispanic whites with AD (O'Bryant et al., 2013c). Furthermore, Bayesian gene-set enrichment identified differential methylation in clusters of genes associated with metabolically driven systemic inflammation in Mexican Americans with AD (Pathak et al., 2019). Additionally, there is an increase in comorbid depression in Mexican Americans with AD (Johnson et al., 2015; Johnson et al., 2019). In this context, changes in the gut microbiome, including those that alter the expression of neurotransmitters and their precursors, have been causally linked to depression in humans (Bastiaanssen et al., 2020; Cruz-Pereira et al., 2020; Yang et al., 2020). Taken together, these studies emphasize how characterization of gut microbiome signature(s) of Americans of Mexican descent with T2D could (Biessels and Whitmer, 2020) provide insight into the relationship between T2D and AD and (Srikanth et al., 2020) identify a new class of innovative treatments to prevent or delay the onset of cognitive impairment in individuals with T2D.

**Figure 1:**
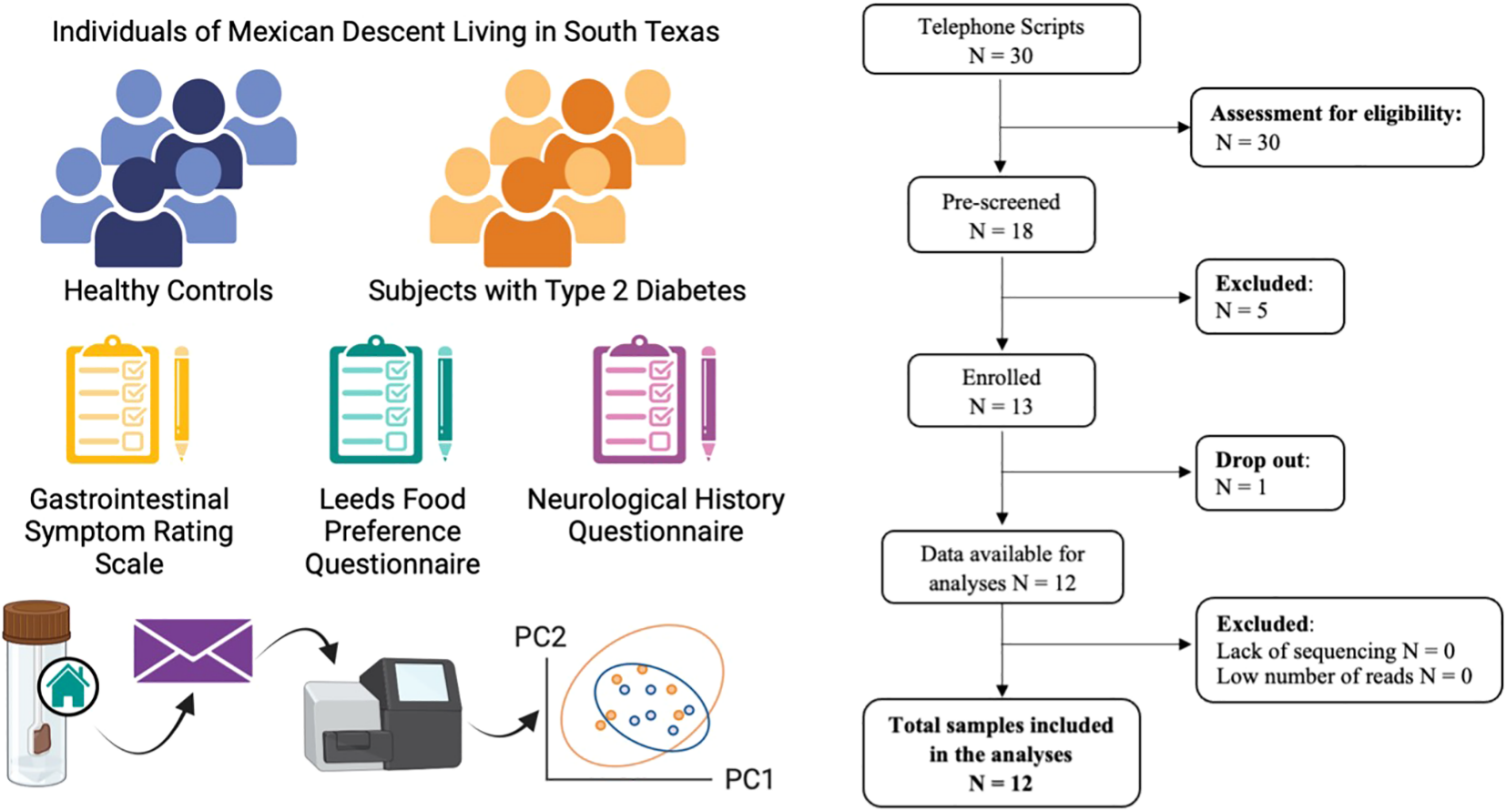
Study schematic and STORMS (Mirzayi et al., 2021) flowchart. Twelve individuals meeting study inclusion criteria completed the study. Each participant completed the gastrointestinal symptom rating scale (GSRS), modified Leeds Food Preference Questionnaire (LFPS), and neurological history questionnaire. Subjects submitted fecal specimens collected at home for 16S ribosomal RNA gene amplicon sequencing and analysis to characterize gut microbial communities.

For this pilot study, we recruited twelve individuals of Mexican descent aged 50 – 70 years living in South Texas, within the Houston-Galveston metroplex and analyzed stool samples along with questionnaire data (**Figure 1**). Half of the study participants were subjects with T2D (sT2D) while the remaining six were healthy controls without diabetes (HC). Here we show that sT2D report a significant increase in gastrointestinal symptom severity compared to HC despite no significant difference in dietary preferences. 16S ribosomal RNA (rRNA) gene amplicon sequencing of stool specimens collected from study participants revealed only slight differences in alpha diversity and a strong interaction of BMI but not diabetes status on community structure. Moreover, we identified associations of specific taxa with diabetes status. Finally, predictive functional profiling identified differentially abundant gene pathways between cohorts. Together, our results suggest that alternations in the functional composition of the gut microbiome of individuals with T2D could precede and potentially contribute to risk for inflammation-associated neurodegenerative disorders.

## Materials and methods

### Recruitment and observational clinical study details

We performed a case-control, cross-sectional observational clinical study based at The University of Texas Medical Branch at Galveston (UTMB). Human subjects were recruited through UTMB’s Endocrinology and Family Medicine clinics in accordance with UTMB Institutional Review Board-approved protocol 20-0201 (ClinicalTrials.gov Identifier NCT04602650; Buffington, PI; Urban, MD) between November 2020 and May 2022. Notably, the study was registered with ClinicalTrials.gov prior to participant recruitment. Subjects were pre-screened to confirm eligibility. Inclusion criteria for sT2D: 50 – 70-year-old males or females of Mexican descent living in Texas with a diagnosis of type 2 diabetes who were willing and able to give informed consent to participate in the study. Inclusion criteria for controls: 50 – 70-year-old males or females of Mexican descent living in Texas without a history of type 2 diabetes who were willing and able to give informed consent to participate in the study. Each group included English-, Spanish-speaking, and bilingual participants. Exclusion Criteria were hypertension requiring more than three anti-hypertensive medications for control, chronic kidney disease (CKD) stage 4 or higher, a history of coronary bypass or stent placement, current pregnancy, and a history of gut inflammation, including irritable bowel syndrome (IBS), celiac disease, or active diverticulitis. Pre-screened subjects that met the defined criteria were consented and enrolled at the UTMB Clinical Research Center. Participants completed medical history, dietary preference, neurological, and gastrointestinal function questionnaires (see **Supplementary File 1**) and were instructed on home use of the provided fecal sample collection kit (DNA GenoTek OMR-200). No subjects were excluded on account of recent antibiotic usage (one subject reported taking a 10-day course of Augmentin roughly 8 weeks prior to sample collection). Subjects were compensated with two gift cards: one upon enrollment and one upon receipt of sample. Samples were de-identified and stored at -80°C until prepared for extraction and analysis. Samples were shipped on dry ice from UTMB in Galveston, TX to Baylor College of Medicine in Houston, TX where they were extracted and analyzed.

### Gastrointestinal Symptom Rating Scale questionnaire and scoring

The GSRS consists of 15 questions to assess reflux (Q2 and 3), abdominal pain (Q1, 4, and 5), indigestion (Q6–9), diarrhea (Q11, 12, and 14), and constipation (Q10, 13, and 15) (Svedlund et al., 1988; Revicki et al., 1998). Subjects were asked to numerically score their subjective symptoms on a scale of 1-7 (1 = no discomfort; 7 = very severe discomfort). The sum of the scores for all 15 items is regarded as the GSRS total score. Total scores ranged from 15 (best outcome) to 105 (worst outcome). The GSRS was administered in the subject’s primary language, English or Spanish. Certified Spanish translation of each questionnaire in the study was provided by UTMB translation services. Cumulative and average scores were calculated. For the average GSRS score, averages of the five categories for subject were averaged and outliers were determined using Grubbs’ test with alpha = 0.05. As data passed the Shapiro-Wilk normality test, but variances were significantly different, statistical significance was determined using an unpaired, two-tailed *t* test with Welch’s correction, where *p<0.05.

### Food preference questionnaire

The food preference questionnaire (FPQ) (Finlayson et al., 2007) screens for known food allergies and food preferences across a variety of categories including red meat, chicken, fish, other protein (e.g., egg), grains and starches, dairy, fruit, vegetables, and sugary or fatty foods. It is a specific 3-item questionnaire in which subjects are asked to first indicate whether they identify as vegan, vegetarian, pescatarian, or none of the above. The subject is then asked whether they have any food allergies to the top eleven common food allergens and given the option to specify a food allergy not listed under, “Other.” Finally, the subject is asked to indicate preference, ranging from, “dislike a lot,” to, “like a lot,” for 59 specific food items listed in tabular format.

### Neurological history questionnaire

The neurological questionnaire is a specific 2-item questionnaire that screens for history of neurological disorders including Alzheimer’s Disease (AD), Parkinson’s Disease (PD), Lewy Body Dementia (LBD), Bipolar Disorder, Schizophrenia, Autism Spectrum Disorder (ASD), Multiple Sclerosis (MS), Amyotrophic Lateral Sclerosis (ALS; Lou Gherig’s Disease), Guillain-Barre Syndrome (GBS), and Attention Deficit and Hyperactivity Disorder (ADHD). In the second part, subjects are asked if they have ever experienced ischemic stroke or mild traumatic brain injury (mTBI).

### Stool sample analysis

Metataxonomic 16S rRNA gene amplicon sequencing was performed by the Baylor College of Medicine Alkek Center for Metagenomics and Microbiome Research (CMMR) as previously described (Sgritta et al., 2019; Buffington et al., 2021; Di Gesu et al., 2022). Briefly, bacterial genomic DNA was extracted using the DNeasy PowerSoil DNA Isolation Kit (MO BIO Laboratories, Carlsbad, CA), and the 16S ribosomal DNA (rDNA) hypervariable region 4 (V4, Forward: GTGCCAGCMGCCGCGGTAA, Reverse: GGACTACHVGGGTWTCTAAT) was amplified by PCR and sequenced on the MiSeq platform (Illumina). A bacterial mock community (MSA-2002™, ATCC) was used as an *in situ* positive control during extraction, amplification, and sequencing of samples. Kit elution buffers and water are used as negative controls during extraction and amplification, respectively. Raw data was uploaded into NCBI BioProject number PRJNA986954. After Trimmomatic (Bolger et al., 2014) and FastQC (Andrews, 2010), reads were imported into R and quality trimmed based on a minimum read length of 50, and truncated to 200–250bp based on quality control scores < 20. Filtered reads were then inferred from Amplicon Sequence Variants (ASVs) using DADA2 (Callahan et al., 2016) followed by chimera removal and taxonomic classification using DECIPHER (Wright, 2016) against the SILVA Database 138 (Quast et al., 2013). ASV counts, taxonomy, and metadata were imported into phyloseq for downstream analysis (McMurdie and Holmes, 2013). ASV counts were agglomerated to genus-level specificity, filtered based on 1% prevalence and detection, and compositionally transformed into relative abundances for taxonomic comparisons and richness estimations. Beta diversity was determined from VST-transformed ASVs based on Euclidian distance in Vegan. The envfit function, part of the vegan package, was used to fit environmental vectors onto an ordination (https://search.r-project.org/CRAN/refmans/vegan/html/envfit.html). MaAsLin2 was used to identify associations between specific ASV/taxa and diabetes status (Mallick et al., 2021).

### Functional inference using PICRUSt2

Functional potential of the microbiome was inferred using PICRUSt2, which predicts the metagenomic content based on ASVs (Douglas et al., 2020). PICRUSt2 was used to predict functional profiles from the normalized count table, identifying functional gene families (MetaCyc and KEGG Pathways) associated with each ASV, and then summing these contributions to obtain the predicted functional profile for each sample. A comparison across diabetes status was conducted using DESeq2 with an upstream independent filtering of pathways containing less than 10 detected counts (Love et al., 2014). Reported results have a log2 fold change>2.5 and an adjusted *p*-value of <0.05. Significance was corrected for multiple comparisons using the Benjamini-Hochberg multiple test correction.

## Results

### Demographics and clinical characteristics

Telephone scripts were read to 30 potential subjects. Of those, 18 were pre-screened. Thirteen subjects were deemed eligible and enrolled. Of those, six healthy controls without diabetes (HC; 1 male, 5 female) and six subjects with type 2 diabetes (sT2D; 3 male, 3 female) completed the study, for a total of 12 participants. The average age of HC and sT2D groups was 54 (HC) and 61 (sT2D) years. Average body mass index (BMI) was 27.9 (HC; range 21.1–34.5) and 34.6 (sT2D; range 28.5–45.4) kg/m^2^, average A1C was 5.3 (HC; range 4.9–5.6) and 7.5 (sT2D; range 5.5–9.6) mg/dL, average systolic pressure was 117 (HC; range 97– 137) and 124 (sT2D; range 106–159) mm Hg, and diastolic pressure was 72.8 (HC; range 58–87) and 79.2 (sT2D; range 72–92) mm Hg (**Supplementary Figure S1**). Only A1C was significantly different between cohorts after correcting for multiple comparisons (**Table 1**). All sT2D were prescribed antidiabetics, including sulfonylureas (glipizide), glucagon-like peptide 1 (GLP-1) receptor agonists (semaglutide), sodium-glucose co-transporter-2 (SGLT-2) inhibitors (empagliflozin), dipeptidyl peptidase-4 (DPP-4) inhibitors (linagliptin), and biguanides (metformin) (**Supplementary Table S1**). All sT2D were taking metformin, and most were on two diabetic medications. No subjects were on insulin, as their non-insulin medications were in the process of titration for dose adjustment to reach maximum dose before adding insulin. Additionally, for some patients, HbA1C targets at 7–8% are appropriate depending on their life expectancy and co-morbidities (Samson et al., 2023).

**Table 1:** Health metrics of participants enrolled in and completing the study.

Characteristic	HC	sT2D	p-value	Adjusted p-value^†^
Age (mean years)	54	61	0.014088	0.070438
Ethnicity, n (%)				
Mexican American	6 (100%)	6 (100%)		
Sex, n (%)				
Female	5 (83%)	3 (50%)		
Male	1 (17%)	3 (50%)		
BMI (mean kg/m^2^ ± SD)	27.9 ± 5.9	34.6 ± 6.7	0.095675	0.478374
A1C (mean mg/dL ± SD)	5.3 ± 0.2	7.5 ± 1.4	0.003162	0.015810^†^
Systolic Pressure (mean mm Hg ± SD)	117.0 ± 15.6	124.0 ± 19.2	0.504296	>0.99999
Diastolic Pressure (mean mm Hg ± SD)	72.8 ± 11.4	79.2 ± 7.2	0.275352	>0.99999

^†^Denotes significance as determined by an unpaired *t* test with Bonferroni-Dunn’s correction for multiple comparisons (FDR Q = 5%).

### Questionnaire results: increased gastrointestinal symptom severity, equivalent food preference reported by sT2D

**Figure 2:**
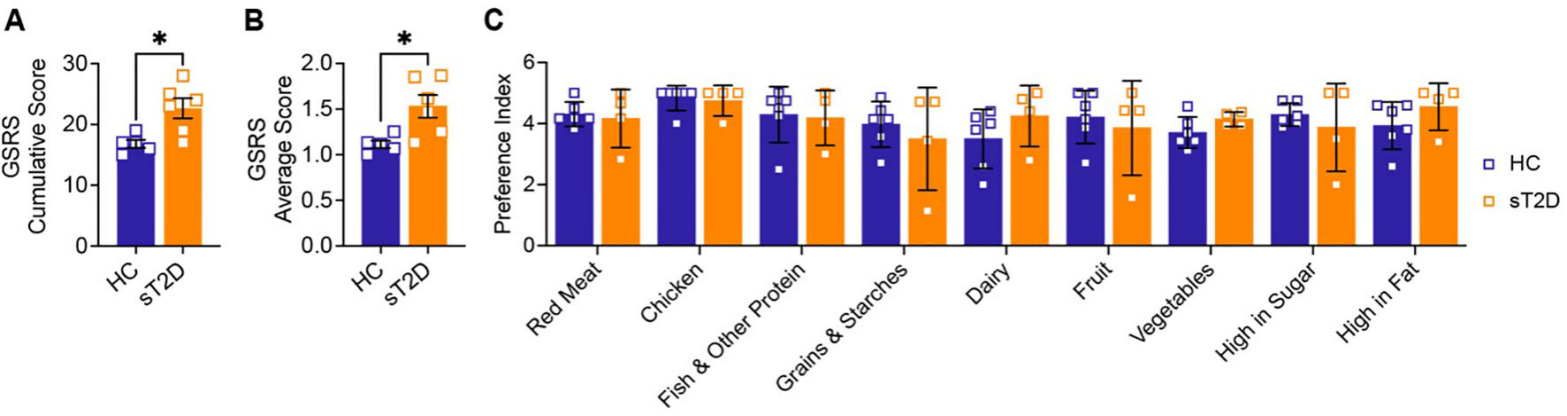
Subjects with Type 2 Diabetes report increased GI symptom severity, but have no change in dietary preferences, compared to healthy controls. **(A)** Cumulative and **(B)** average severity scores from the gastrointestinal symptom rating scale (GSRS) show that sT2D report higher GI symptom severity compared to HC [A: (t(6.539) = 3.305, *p* = 0.0144); B: (t(6.073) = 3.187, *p* = 0.0186] as determined by a two-tailed unpaired Welch’s *t*-test, with one outlier removed (Grubbs’ test where alpha = 0.05; *p < 0.05). **(C)** In contrast, food preferences are not statistically different between groups. Subjects were asked to rank foods by preference, where 0 = Not applicable, 1 = Dislike a lot, 2 = Dislike a little, 3 = Neither like nor dislike, 4 = Like a little, and 5 = Like a lot. Analysis by a mixed-effects model with Šídák’s correction for multiple comparisons did not identify any significant differences between HC and sT2D.

No neurological events (see Materials and Methods) were reported by study participants. Interestingly, both cumulative and category-averaged GSRS scores were significantly higher in sT2D compared to HCs (**Figures 2A, B**), despite no differences in dietary expressed preferences (**Figure 2C**). No significant differences among individual GSRS categories (reflux, abdominal pain, indigestion, diarrhea, or constipation) were identified between groups (**Supplementary Figure S2**).

### Microbiome analysis reveals that BMI is a significant contributor to gut community structure

**Figure 3:**
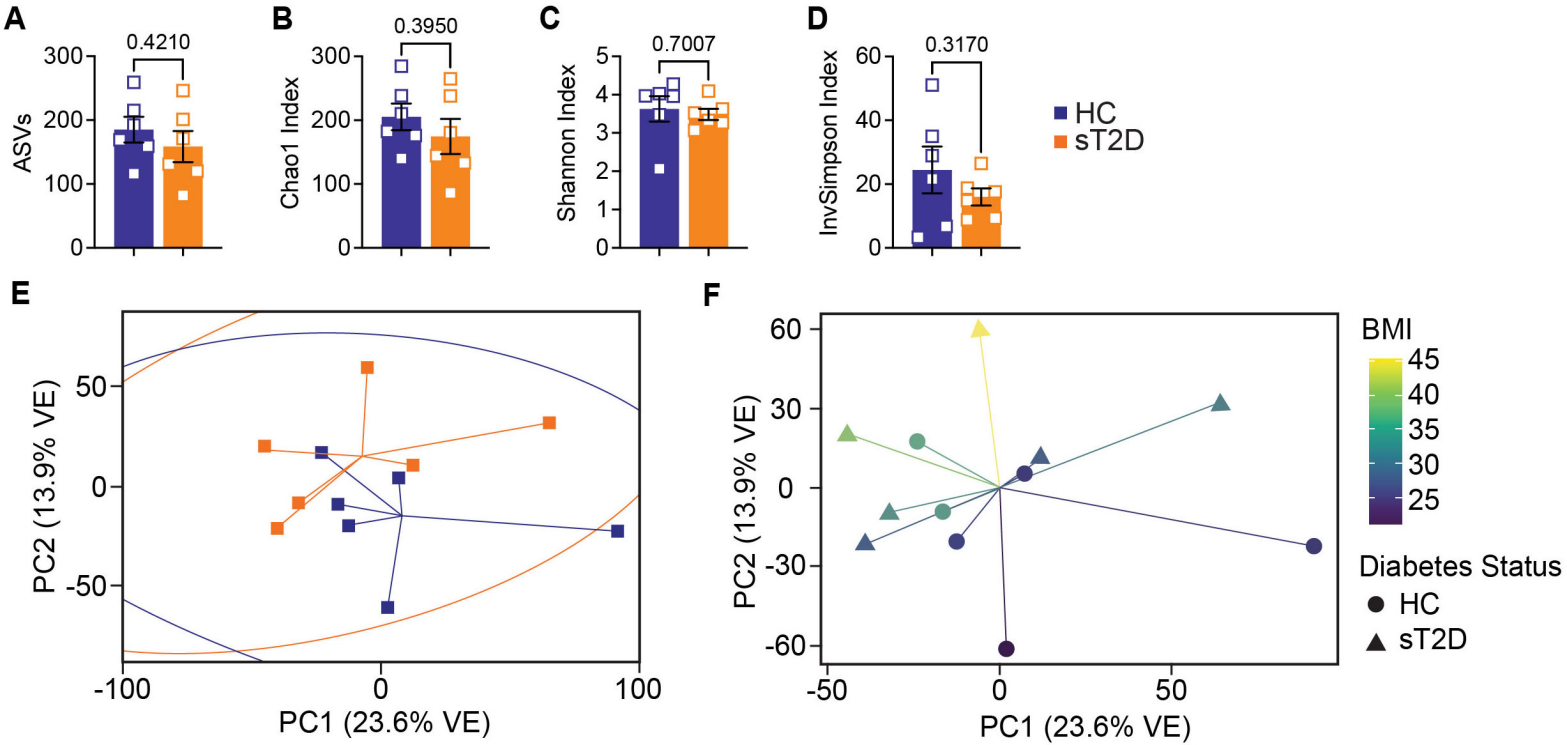
Analysis of alpha diversity metrics among study participants revealed a trending decrease in microbial diversity among subjects with T2D. **(A–D)** Alpha diversity graphs for males and females combined analyzed by two-tailed, unpaired Welch’s t-test: **(A)** ASVs (*t*(9.661) = 0.8404, p = 0.4210), **(B)** Chao1 Index (*t*(9.323) = 0.8917, p = 0.395), **(C)** Shannon Index (*t*(6.877) = 0.4008, p = 0.7007), and **(D)** Inverse Simpson Index (*t*(6.328) = 1.086, p = 0.317). Principal coordinate analysis of **(E)** Bray-Curtis dissimilarities analyzed by permutational ANOVA (p = 0.175, R^2^ = 0.1028, permutations = 999) did not reveal statistically significant clusters between subject groups but did trend toward significance based on **(F)** BMI values (p = 0.0740, R^2^ = 0.1188, permutations = 999). Similarly, unweighted UniFrac (which incorporates phylogenetic distance but does not consider abundance) analysis was significantly different for BMI but not Diabetes Status; however, this significance was not observed by Weighted UniFrac (which does consider abundance). Importantly, betadisper analysis did not reveal significant *within group* significant differences by either method (Bray-Curtis: p = 0.7844; Unweighted and Weighted UniFrac: p = 0.1512 and 0.9136, respectively). See also **Supplementary File 2**.

To determine if any differences were present at the genus level between sT2D and HC, we performed metataxonomic 16S ribosomal RNA (rRNA) gene amplicon sequencing of stool samples provided by study participants (see Materials & Methods). Although averages for combined alpha (within sample) diversity metrics (**Figures 3A–D**) including amplicon sequence variants (ASVs), Chao1 index, Shannon Index, and Inverse Simpson Index were lower for sT2D compared to HC samples, they were not statistically significant. Furthermore, we observed no significant differences in beta (between sample) diversity as measured by permutational ANOVA of Bray-Curtis (**Figure 3E**), and Weighted (Diabetes Status: p = 0.5682, R^2^ = 0.0904, permutations = 9999; BMI: p = 0.8771, R^2^ = 0.0159, permutations = 9999) or Unweighted UniFrac distances (Diabetes Status: p = 0.6119, R^2^ = 0.0780, permutations = 9999; BMI: p = 0.0121, R^2^ = 0.1470, permutations = 9999), possibly due to the small sample size of this pilot cohort. However, sample clusters did trend towards significance based on body mass index (BMI) values (**Figure 3F**). Specifically, use of the Envfit function to fit environmental vectors age and BMI onto an ordination plot revealed that BMI, but not age, is a significant driver of community structure (BMI: p = 0.007, R^2^ = 0.7062, permutations = 999; Age: p = 0.750, R^2^ = 0.0548, permutations = 999; **Supplementary Figure S3**).

### Microbiome analysis reveals significant differences in taxa abundance between groups

**Figure 4:**
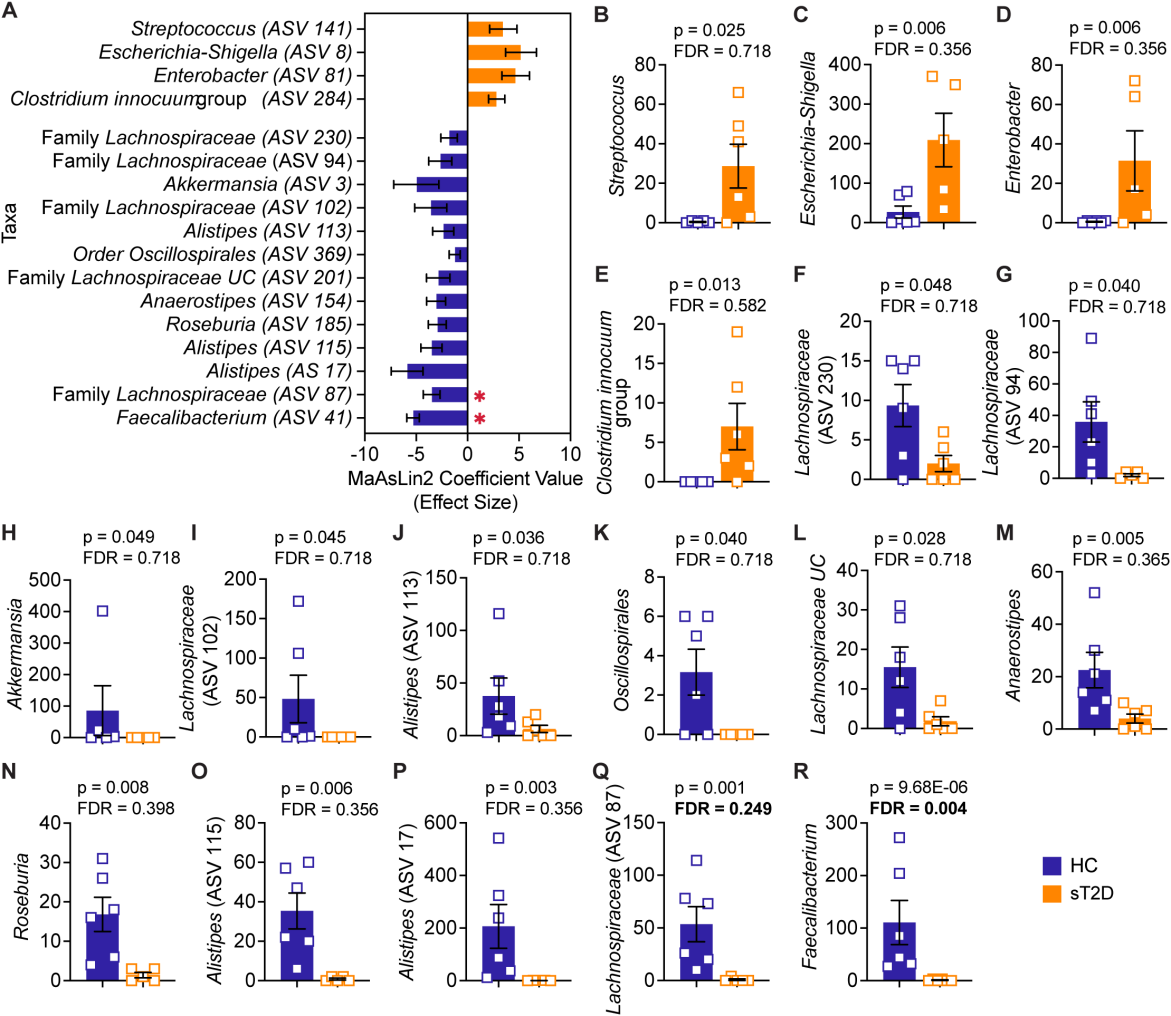
Microbiome Multivariable Associations with Linear Models (MaAsLin2) detected associations of specific taxa with diabetes status. **(A)** Histogram of MaAsLin2 Coefficient Values (effect sizes) for genus-level taxa with p < 0.05. *Indicates significance after FDR correction. **(B–R)** ASV counts by subject group for **(B)** *Streptococcus*, **(C)** *Escherichia-Shigella*, **(D)** *Enterobacter*, **(E)** *Clostridium innocuum* group, **(F)** *Lachnospiraceae* (ASV 230), **(G)** *Lachnospiraceae* (ASV 94), **(H)** *Akkermansia*, **(I)** *Lachnospiraceae* (ASV 102), **(J)** *Alistipes* (ASV 113), **(K)** *Oscillospirales*, **(L)** *Lachnospiraceae* UC, **(M)** *Anaerostipes*, **(N)** *Roseburia*, **(O)** *Alistipes* (ASV 115), **(P)** *Alistipes* (ASV 17), **(Q)** *Lachnospiraceae* (ASV 87), and **(R)** *Faecalibacterium*, with MaAsLin2 p- and q-values shown above each taxa. UC; uncultured.

We next used Microbiome Multivariable Associations with Linear Models (MaAsLin2) (Mallick et al., 2021) to determine if any associations existed between ASVs and diabetes status, and identified seventeen taxa with p<0.05 (**Figure 4A**, **Supplementary File 2**), of which two passed after controlling for false discovery (*Faecalibacterium*, FDR=0.003; *Lachnospiraceae*, FDR=0.249). sT2D samples were associated with *Streptococcus, Escherichia-Shigella, Enterobacter*, and *Clostridum innocuum* (**Figures 4B–E**), while HC samples were associated with *Lachnospiraceae*, *Oscillobacter*, *Akkermansia*, *Alistipes*, *Anaerostipes*, *Roseburia*, and *Faecalibacterium* (**Figures 4F–R**).

### Predictive functional profiling identifies differentially abundant gene pathways between cohorts

**Figure 5:**
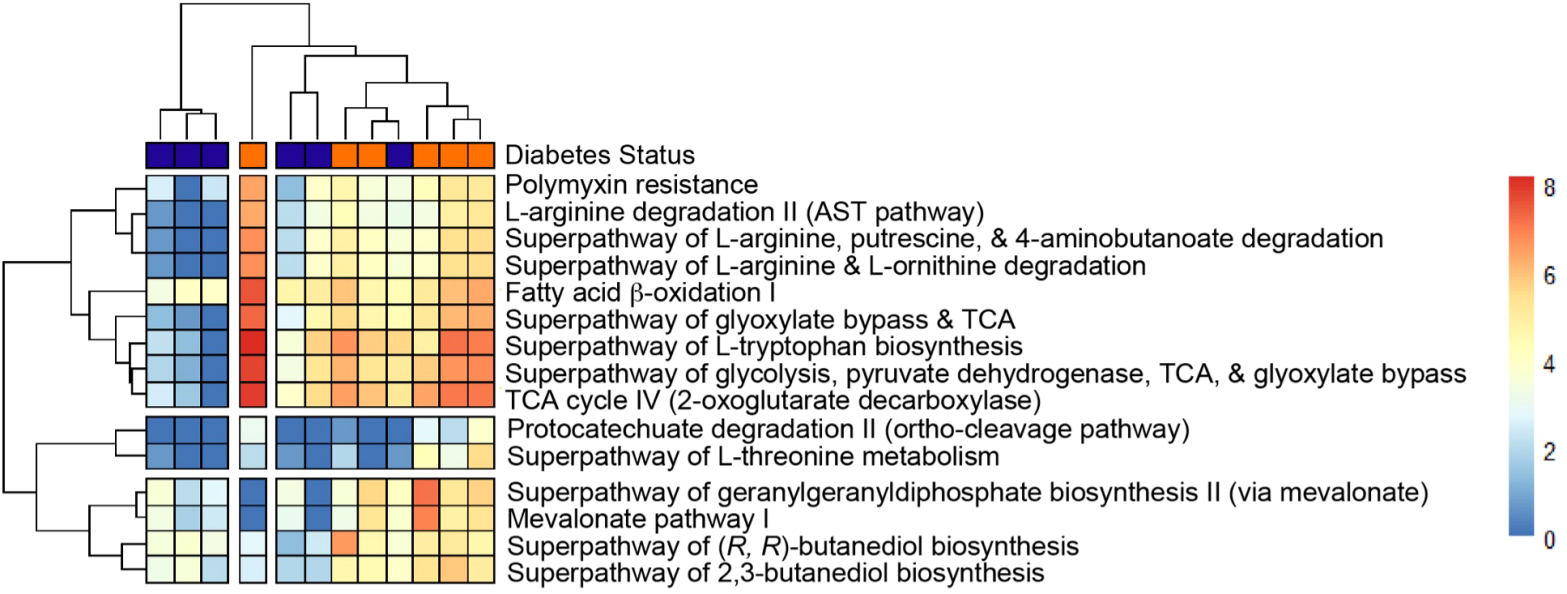
Heatmap of statistically significant Metacyc features that differ between groups with p < 0.05. Heatmap from Metacyc metabolic database based on PICRUSt2 output (See Materials and Methods and **Table 3** for p-values).

Finally, to perform predictive functional profiling, we first used PICRUSt2 to generate functional outputs based on Kyoto Encyclopedia of Genes and Genomes (KEGG) (Goto et al., 1997; Kanehisa et al., 2012) and MetaCyc databases (**Tables 2**, **3**, **Figure 5**), and then performed pairwise comparisons using DESeq2 to determine if any of these pathways were significantly enriched in sT2D compared to HC samples. We identified four significantly differentially abundant KEGG pathways: Bacterial invasion of epithelial cells, *Staphylococcus aureus* infection, alpha-linolenic acid metabolism, and polycyclic aromatic hydrocarbon degradation (**Table 2**). Twenty-five KEGG orthologs (KO) present in 105 ASVs contributed to these pathways (**Supplementary Table S2**, **Supplementary File 3**). From MetaCyc, we identified 15 significantly differentially abundant metabolic pathways (**Table 3**, **Figure 5**).

**Table 2:** Results of four significant differentially abundant ASV-inferred KEGG pathways associated with sT2D compared to HC samples (see also **Supplementary Table S2**, **Supplementary File 3**).

Feature	Pathway Name	Log_2_FoldChange	Adjusted p-value
KO00592	Alpha-linolenic acid metabolism	2.03	0.0066
KO00624	Polycyclic aromatic hydrocarbon degradation	9.70	0.0034
KO05150	Staphylococcus aureus infection	2.90	0.0001
KO05100	Bacterial invasion of epithelial cells	3.28	0.0074

**Table 3:** Results of 15 significant differentially abundant ASV-inferred MetaCyc metabolic pathways associated with sT2D compared to HC samples (See also **Figure 5**).

Pathway	Description	Log_2_FoldChange	Adjusted p-value
ARGDEG-PWY	Superpathway of L-arginine, putrescine, & 4-aminobutanoate degradation	3.887	0.049
AST-PWY	L-arginine degradation II	3.919	0.049
FAO-PWY	Fatty acid β-oxidation I	2.806	0.019
GLYCOLYSIS-TCA-GLYOX-BYPASS	Superpathway of glycolysis, pyruvate dehydrogenase, TCA, & glyoxylate bypass	3.561	0.036
ORNARGDEG-PWY	Superpathway of L-arginine & L-ornithine degradation	3.887	0.049
P105-PWY	TCA cycle IV (2-oxoglutarate decarboxylase)	3.686	0.029
P125-PWY	Superpathway of (*R,R*)-butanediol biosynthesis	2.873	0.048
PROTOCATECHUATE-ORTHO-CLEAVAGE-PWY	Protocatechuate degradation II (ortho-cleavage pathway)	6.595	0.049
PWY-5910	Superpathway of geranylgeranyldiphosphate biosynthesis I (via mevalonate)	3.911	0.049
PWY-6396	Superpathway of 2,3-butanediol biosynthesis	2.865	0.049
PWY-6629	Superpathway of L-tryptophan biosynthesis	3.319	0.049
PWY-922	Mevalonate pathway I	3.978	0.049
PWY0-1338	Polymyxin resistance	3.374	0.049
TCA-GLYOX-BYPASS	Superpathway of glyoxylate bypass & TCA	3.750	0.036
THREOCAT-PWY	Superpathway of L-threonine metabolism	7.310	0.014

## Discussion

A growing body of literature links inflammation and dysbiosis of the gut microbiome to AD susceptibility [for review see (Stolzer et al., 2023)]. For instance, a recent study of a small cohort of dementia patients revealed divergent gut microbiota composition, increased gut permeability, and inflammation, implicating a microbial determinant in neuroimmune dysregulation, an emerging player in neurodegeneration (Stadlbauer et al., 2020). Specifically, authors reported increased serum diaminooxidase (DAO) and soluble CD-14, markers of intestinal permeability and inflammation, respectively, in subjects with dementia. Although alpha diversity was unchanged, the authors did report divergent gut ecology (as determined by beta diversity analysis) in subjects with dementia compared to controls that also clustered by severity. The aim of this current study was to examine if changes in gut community structure occur in individuals with diabetes within a population at increased risk for AD. This multidisciplinary pilot study builds on previous investigations into the gut microbial community structure of a similar cohort of Americans of Mexican descent with high rates of obesity and diabetes (Fisher-Hoch et al., 2010; Ross et al., 2015). Although not statistically significant, our finding that some metrics of within-sample (alpha) diversity were slightly lower in gut microbiomes of sT2D compared to HC (**Figures 3A, B, D**) agrees with a previous study reporting altered composition and functional capacity of gut microbiomes in obese patients (Thingholm et al., 2019). This same study, which examined the gut microbiomes of hundreds of lean non-diabetic, obese non-diabetic, and obese type 2 diabetic subjects, also reported differences in between-sample (beta) diversity not captured in our small pilot study. Importantly, however, our findings agree with other previously reported associations between T2D or other chronic conditions (including inflammatory bowel disease) and changes in specific taxa, including higher abundance of *Escherichia-Shigella* and lower abundance of *Faecalibacterium*, *Alistipes*, *Oscillobacter*, *Roseburia*, and *Akkermansia* (**Figure 4**) (Thingholm et al., 2019; Mallick et al., 2021). Given the person-to-person variability observed among gut microbiome profiles of aging populations (Claesson et al., 2011), our results from a small cohort could be indicative of a predictive disease signature among patients with T2D predisposed toward AD. Furthermore, they support a hypothesis that a subgroup of patients with type 2 diabetes who would go on to develop AD could benefit from characterization and precision targeting of the gut microbiome to reduce inflammation and thereby prevent or at least postpone the onset of dementia.

Our study is not without limitations. First, enrollment took place from 2020–2022, and recruitment challenges associated with the COVID-19 pandemic resulted in a small sample size (N =12). Longitudinal, sufficiently powered studies are needed to continue exploring how alterations in gut microbiome community structure contribute to risk for neurodegenerative disorders. Second, while we did not detect any differences in food preferences between HC and sT2D, it is possible that the food preference questionnaire – although appropriate to assess exposure to a Western pattern diet high in carbohydrate and fat but low in fiber content – was not ideally suited to our specific cohort. Additionally, we do not have any data on the immigration history of participants, which might inform shifts in diet and therefore microbiome composition. Of note, all six sT2D were prescribed metformin, a known modulator of gut microbiome community composition (Sun et al., 2018), and treatment with sulfonylurea drugs (taken by one subject here) increases risk for hypoglycemia, which may accelerate dementia (Meneilly and Tessier, 2016). Notably, Thingholm et al. found clear associations between host microbiome variation and medications, supplementations, and diet, making direct changes due to diabetes status difficult to establish. Therefore, the potentially confounding variable of medication (including antihypertensive and antilipemic drugs) should be considered in subsequent studies examining links between the gut microbiome, diabetes, and dementia. Also of note, in our study BMI was not significantly different between cohorts (*p* = 0.096144, **Table 1**) but did strongly associate with community structure (**Table 1**, **Figure 3**, **Supplementary Figure S3**). We therefore found it curious that BMI, but not diabetes, is a strong driver of gut community structure. Given the findings of Thingholm et al., it is possible that the gut-modulating medications being taken by the subjects with T2D could mask an associated risk/contribution to gut ecology, whereas BMI is an indicator of obesity which is driven by diet, also the main driver of gut microbiome ecologyx. Of note, no healthy control subjects were pre-diabetic based on A1C, but there is an overlap in the range of BMIs across groups. Notably, it is difficult to match BMI between cohorts in a diabetes study, given that a BMI ≥ 30 kg/m^2^ has been shown to be a strong indicator of “adult lifetime risk of diabetes (Narayan et al., 2007)” nevertheless, the role of diabetes specifically (independent of obesity) as a driver of AD must be carefully teased out in future work. A technical limitation of this work is that amplicon-based sequencing provides genus-level resolution, whereas metagenomic whole genome shotgun sequencing of participant samples would provide species-to-strain level-specific information as well as functional data (as opposed to predicted functions which may not capture “rare environment-specific functions (Douglas et al., 2020)” of bacteria whose genomes are not currently well-represented). Indeed, metagenomic combined with metatranscriptomic analysis of gut microbiota from patients with T2D both pre- and post-medication, and post-AD diagnosis, will be critical to differentiate between changes in the composition and functional profile of the gut microbiota due to various medications and those that may directly contribute to neurodegeneration. Lastly, given the small sample size and only one male in our control cohort, we could not evaluate sex differences. However, future studies should examine sex differences, which may be relevant given that that 1) women are disproportionately affected by AD and 2) recently published results in preclinical animal models performed by our lab show a sexually dimorphic impact of diet and [probiotic] supplementation alike on host gut microbiota composition (Di Gesu et al., 2022).

Data presented here adds to the growing body of work seeking to determine how T2D predisposes individuals to the cognitive impairment and underlying neuropathology characteristic of AD. Given our findings that sT2D reported increase gastrointestinal symptoms compared to HC (**Figure 2**), and previous reports of increased gut permeability and endotoxin load, a noteworthy ASV-inferred functional KEGG pathway that was differentially abundant in sT2D in our study was the bacterial invasion of epithelial cells (pathway ko05100, **Table 2**, **Supplementary Table S2**, **Supplementary File 3**). Consistent with this finding, we observed a significant association of *Faecalibacterium* among healthy control gut microbiota (**Figure 4**). Notably, *Faecalibacterium prausnitzii* has been shown to enhance gut barrier integrity and intestinal immunity (Al-Fakhrany and Elekhnawy, 2024). Thus, future studies examining gut permeability and gut barrier integrity, as well as the therapeutic potential of probiotic strains with capacity to strengthen gut barrier integrity, in T2D patient populations predisposed to AD may be warranted. Conversely, the microbiome is also a source of neuroprotective metabolites, such as indole derivatives which modulate host inflammation (Pappolla et al., 2021). Surprisingly, differentially abundant ASV-inferred MetaCyc metabolic pathways (including the tryptophan pathway, an indole precursor) were all elevated in sT2D compared to HC (**Table 3**, **Figure 5**). Although many factors, both genetic and environmental, contribute to AD susceptibility, targeting the host gut microbiome may reduce risk in a predisposed population, as well as in a proportion of the population at large. Exemplifying this is work in our lab showing striking improvements in offspring neurological outcomes simply by altering the maternal environment (gut and serum) through probiotics. As this study was performed in a very specific demographic, future work comparing our results to other minority populations at higher risk of T2D would help understand the impact of environment (diet, lifestyle, etc.), genetics, and their interactions on AD risk. For instance, given that we found BMI to be a major driver of microbiome community structure among participants in our study (**Figure 3F**), it is possible that the mechanisms at play and associated risk profile would be similar to those impacting populations disproportionately affected by obesity (Hales et al., 2018; Liu et al., 2021).

The results of this study bring us one step closer to the identification of a microbial or microbially associated signature that predicts dementia risk, as well as pre- or probiotics that can modulate said signatures, which would be a key breakthrough that could revolutionize care for patients with metabolic and neurodegenerative disorders alike. Moreover, it highlights the therapeutic potential of targeting the gut microbiome to dampen neuroinflammation in the context of metabolic and neurodegenerative disorders.

## Data Availability

The datasets presented in this study can be found in online repositories. The names of the repository/repositories and accession number(s) can be found below: https://www.ncbi.nlm.nih.gov/, PRJNA986954. Deidentified primary data will be made available upon request. R code for the microbiome analysis in **Figure 3**, **Figure 4**, **Supplementary Figure S3**, **Table 2**, **Table 3**, and **Supplementary Table S2** is available at https://github.com/MADscientist314/lmatz. R code for the MaAsLin2 analysis in **Figure 4** is available at https://github.com/lisamatz/Matz-et-al.-gut-pathobionts. A STORMS (Strengthening The Organizing and Reporting of Microbiome Studies) (69) checklist is available at 10.5281/zenodo.10723337.

## References

[B1] Al-Fakhrany O. M. Elekhnawy E. (2024). Next-generation probiotics: the upcoming biotherapeutics. Mol. Biol. Rep. 51, 505. doi: 10.1007/s11033-024-09398-5 38619680 PMC11018693

[B2] Andrews S. (2010). FastQC: a quality control tool for highthroughput sequence data. Available online at: https://www.bioinformatics.babraham.ac.uk/projects/fastqc/.

[B3] Bastiaanssen T. F. S. Cussotto S. Claesson M. J. Clarke G. Dinan T. G. Cryan J. F. (2020). Gutted! Unraveling the role of the microbiome in major depressive disorder. Harv Rev. Psychiatry 28, 26–39. doi: 10.1097/HRP.0000000000000243 31913980 PMC7012351

[B4] Berntzen B. J. Jukarainen S. Bogl L. H. Rissanen A. Kaprio J. Pietilainen K. H. (2019). Eating behaviors in healthy young adult twin pairs discordant for body mass index. Twin Res. Hum. Genet. 22, 220–228. doi: 10.1017/thg.2019.43 31466550

[B5] Biessels G. J. Despa F. (2018). Cognitive decline and dementia in diabetes mellitus: mechanisms and clinical implications. Nat. Rev. Endocrinol. 14, 591–604. doi: 10.1038/s41574-018-0048-7 30022099 PMC6397437

[B6] Biessels G. J. Whitmer R. A. (2020). Cognitive dysfunction in diabetes: how to implement emerging guidelines. Diabetologia. 63, 3–9. doi: 10.1007/s00125-019-04977-9 31420699 PMC6890615

[B7] Bolger A. M. Lohse M. Usadel B. (2014). Trimmomatic: a flexible trimmer for Illumina sequence data. Bioinformatics. 30, 2114–2120. doi: 10.1093/bioinformatics/btu170 24695404 PMC4103590

[B8] Buffington S. A. Dooling S. W. Sgritta M. Noecker C. Murillo O. D. Felice D. F.. (2021). Dissecting the contribution of host genetics and the microbiome in complex behaviors. Cell. 184, 1740–56.e16. doi: 10.1016/j.cell.2021.02.009 33705688 PMC8996745

[B9] Callahan B. J. McMurdie P. J. Rosen M. J. Han A. W. Johnson A. J. A. Holmes S. P. (2016). DADA2: High-resolution sample inference from Illumina amplicon data. Nat. Methods 13, 581–583. doi: 10.1038/nmeth.3869 27214047 PMC4927377

[B10] Center for Disease Control and Prevention (2018). Division of nutrition, physical activity, and obesity. Data Trend Maps. NCfCDPaHP. Available online at: https://www.cdc.gov/nccdphp/dnpao/data-trends-maps/index.html (Accessed November 18, 2024).

[B11] Claesson M. J. Cusack S. O'Sullivan O. Greene-Diniz R. de Weerd H. Flannery E.. (2011). Composition, variability, and temporal stability of the intestinal microbiota of the elderly. Proc. Natl. Acad. Sci. U S A. 108 Suppl 1, 4586–4591. doi: 10.1073/pnas.1000097107 20571116 PMC3063589

[B12] Clemente J. C. Ursell L. K. Parfrey L. W. Knight R. (2012). The impact of the gut microbiota on human health: an integrative view. Cell. 148, 1258–1270. doi: 10.1016/j.cell.2012.01.035 22424233 PMC5050011

[B13] Cruz-Pereira J. S. Rea K. Nolan Y. M. O'Leary O. F. Dinan T. G. Cryan J. F. (2020). Depression's unholy trinity: dysregulated stress, immunity, and the microbiome. Annu. Rev. Psychol. 71, 49–78. doi: 10.1146/annurev-psych-122216-011613 31567042

[B14] David L. A. Maurice C. F. Carmody R. N. Gootenberg D. B. Button J. E. Wolfe B. E.. (2014). Diet rapidly and reproducibly alters the human gut microbiome. Nature. 505, 559–563. doi: 10.1038/nature12820 24336217 PMC3957428

[B15] Di Gesu C. M. Matz L. M. Bolding I. J. Fultz R. Hoffman K. L. Gammazza A. M.. (2022). Maternal gut microbiota mediate intergenerational effects of high-fat diet on descendant social behavior. Cell Rep. 41, 111461. doi: 10.1016/j.celrep.2022.111461 36223744 PMC9597666

[B16] Douglas G. M. Maffei V. J. Zaneveld J. R. Yurgel S. N. Brown J. R. Taylor C. M.. (2020). PICRUSt2 for prediction of metagenome functions. Nat. Biotechnol. 38, 685–688. doi: 10.1038/s41587-020-0548-6 32483366 PMC7365738

[B17] Dove A. Dunk M. M. Wang J. Guo J. Whitmer R. A. Xu W. (2024). Anti-inflammatory diet and dementia in older adults with cardiometabolic diseases. JAMA Netw. Open 7, e2427125. doi: 10.1001/jamanetworkopen.2024.27125 39133488 PMC11320167

[B18] Finlayson G. King N. Blundell J. E. (2007). Liking vs. wanting food: importance for human appetite control and weight regulation. Neurosci. Biobehav. Rev. 31, 987–1002. doi: 10.1016/j.neubiorev.2007.03.004 17559933

[B19] Fisher-Hoch S. P. Rentfro A. R. Salinas J. J. Pérez A. Brown H. S. Reininger B. M.. (2010). Socioeconomic status and prevalence of obesity and diabetes in a Mexican American community, Cameron County, Texas, 2004-2007. Prev. Chronic Dis. 7, A53. Available online at: http://www.cdc.gov/pcd/issues/2010/may/09_0170.htm (Accessed November 18, 2024). 20394692 PMC2879985

[B20] Flegal K. M. Kruszon-Moran D. Carroll M. D. Fryar C. D. Ogden C. L. (2016). Trends in obesity among adults in the United States, 2005 to 2014. JAMA. 315, 2284–2291. doi: 10.1001/jama.2016.6458 27272580 PMC11197437

[B21] Franks P. W. McCarthy M. I. (2016). Exposing the exposures responsible for type 2 diabetes and obesity. Science. 354, 69–73. doi: 10.1126/science.aaf5094 27846494

[B22] Goto S. Bono H. Ogata H. Fujibuchi W. Nishioka T. Sato K.. (1997). Organizing and computing metabolic pathway data in terms of binary relations. Pac Symp Biocomput, 175–186. 9390290

[B23] Guzman-Castaneda S. J. Ortega-Vega E. L. de la Cuesta-Zuluaga J. Velasquez-Mejia E. P. Rojas W. Bedoya G.. (2020). Gut microbiota composition explains more variance in the host cardiometabolic risk than genetic ancestry. Gut Microbes 11, 191–204. doi: 10.1080/19490976.2019.1634416 31311405 PMC7053924

[B24] Hales C. M. Fryar C. D. Carroll M. D. Freedman D. S. Aoki Y. Ogden C. L. (2018). Differences in obesity prevalence by demographic characteristics and urbanization level among adults in the United States, 2013-2016. JAMA. 319, 2419–2429. doi: 10.1001/jama.2018.7270 29922829 PMC6583043

[B25] Jasarevic E. Bale T. L. (2019). Prenatal and postnatal contributions of the maternal microbiome on offspring programming. Front. Neuroendocrinol. 55, 100797. doi: 10.1016/j.yfrne.2019.100797 31574280

[B26] Johnson L. A. Gamboa A. Vintimilla R. Cheatwood A. J. Grant A. Trivedi A.. (2015). Comorbid depression and diabetes as a risk for mild cognitive impairment and alzheimer's disease in elderly Mexican Americans. J. Alzheimers Dis. 47, 129–136. doi: 10.3233/JAD-142907 26402761 PMC13270981

[B27] Johnson L. A. Large S. E. Izurieta Munoz H. Hall J. R. O'Bryant S. E. (2019). Vascular depression and cognition in Mexican Americans. Dement Geriatr. Cognit. Disord. 47, 68–78. doi: 10.1159/000494272 30861514

[B28] Kanehisa M. Goto S. Sato Y. Furumichi M. Tanabe M. (2012). KEGG for integration and interpretation of large-scale molecular data sets. Nucleic Acids Res. 40, D109–D114. doi: 10.1093/nar/gkr988 22080510 PMC3245020

[B29] Lillioja S. Mott D. M. Spraul M. Ferraro R. Foley J. E. Ravussin E.. (1993). Insulin resistance and insulin secretory dysfunction as precursors of non-insulin-dependent diabetes mellitus. Prospective Stud. Pima Indians. N Engl. J. Med. 329, 1988–1992. doi: 10.1056/NEJM199312303292703 8247074

[B30] Liu B. Du Y. Wu Y. Snetselaar L. G. Wallace R. B. Bao W. (2021). Trends in obesity and adiposity measures by race or ethnicity among adults in the United States 2011-18: population based study. BMJ 372, n365. doi: 10.1136/bmj.n365 33727242 PMC7961695

[B31] Longoria C. R. Guers J. J. Campbell S. C. (2022). The interplay between cardiovascular disease, exercise, and the gut microbiome. Rev. Cardiovasc. Med. 23, 365. doi: 10.31083/j.rcm2311365 39076202 PMC11269073

[B32] Love M. I. Huber W. Anders S. (2014). Moderated estimation of fold change and dispersion for RNA-seq data with DESeq2. Genome Biol. 15, 550. doi: 10.1186/s13059-014-0550-8 25516281 PMC4302049

[B33] Mallick H. Rahnavard A. McIver L. J. Ma S. Zhang Y. Nguyen L. H.. (2021). Multivariable association discovery in population-scale meta-omics studies. PLoS Comput. Biol. 17, e1009442. doi: 10.1371/journal.pcbi.1009442 34784344 PMC8714082

[B34] Mambiya M. Shang M. Wang Y. Li Q. Liu S. Yang L.. (2019). The play of genes and non-genetic factors on type 2 diabetes. Front. Public Health 7, 349. doi: 10.3389/fpubh.2019.00349 31803711 PMC6877736

[B35] McMurdie P. J. Holmes S. (2013). phyloseq: an R package for reproducible interactive analysis and graphics of microbiome census data. PLoS One 8, e61217. doi: 10.1371/journal.pone.0061217 23630581 PMC3632530

[B36] Meneilly G. S. Tessier D. M. (2016). Diabetes, dementia and hypoglycemia. Can. J. Diabetes. 40, 73–76. doi: 10.1016/j.jcjd.2015.09.006 26778684

[B37] Mirzayi C. Renson A. Furlanello C. Sansone S. A. Zohra F. Elsafoury S.. (2021). Reporting guidelines for human microbiome research: the STORMS checklist. Nat. Med. 27, 1885–1892. doi: 10.1038/s41591-021-01552-x 34789871 PMC9105086

[B38] Mozaffarian D. Hao T. Rimm E. B. Willett W. C. Hu F. B. (2011). Changes in diet and lifestyle and long-term weight gain in women and men. N Engl. J. Med. 364, 2392–2404. doi: 10.1056/NEJMoa1014296 21696306 PMC3151731

[B39] Narayan K. M. Boyle J. P. Thompson T. J. Gregg E. W. Williamson D. F. (2007). Effect of BMI on lifetime risk for diabetes in the U. S. Diabetes Care 30, 1562–1566. doi: 10.2337/dc06-2544 17372155

[B40] Nicholson J. K. Holmes E. Kinross J. Burcelin R. Gibson G. Jia W.. (2012). Host-gut microbiota metabolic interactions. Science. 336, 1262–1267. doi: 10.1126/science.1223813 22674330

[B41] O'Bryant S. E. Johnson L. Balldin V. Edwards M. Barber R. Williams B.. (2013a). Characterization of Mexican Americans with mild cognitive impairment and Alzheimer's disease. J. Alzheimers Dis. 33, 373–379. doi: 10.3233/JAD-2012-121420 22976076 PMC3524411

[B42] O'Bryant S. E. Johnson L. Reisch J. Edwards M. Hall J. Barber R.. (2013b). Risk factors for mild cognitive impairment among Mexican Americans. Alzheimers Dement. 9, 622–31.e1. doi: 10.1016/j.jalz.2012.12.007 23643456 PMC3737282

[B43] O'Bryant S. E. Xiao G. Edwards M. Devous M. Gupta V. B. Martins R.. (2013c). Biomarkers of Alzheimer's disease among Mexican Americans. J. Alzheimers Dis. 34, 841–849. doi: 10.3233/JAD-122074 23313927 PMC3608404

[B44] Pappolla M. A. Perry G. Fang X. Zagorski M. Sambamurti K. Poeggeler B. (2021). Indoles as essential mediators in the gut-brain axis. Their role in Alzheimer's disease. Neurobiol. Dis. 156, 105403. doi: 10.1016/j.nbd.2021.105403 34087380

[B45] Pathak G. A. Silzer T. K. Sun J. Zhou Z. Daniel A. A. Johnson L.. (2019). Genome-wide methylation of mild cognitive impairment in Mexican Americans highlights genes involved in synaptic transport, alzheimer's disease-precursor phenotypes, and metabolic morbidities. J. Alzheimers Dis. 72, 733–749. doi: 10.3233/JAD-190634 31640099

[B46] Quast C. Pruesse E. Yilmaz P. Gerken J. Schweer T. Yarza P.. (2013). The SILVA ribosomal RNA gene database project: improved data processing and web-based tools. Nucleic Acids Res. 41, D590–D596. doi: 10.1093/nar/gks1219 23193283 PMC3531112

[B47] Revicki D. A. Wood M. Wiklund I. Crawley J. (1998). Reliability and validity of the Gastrointestinal Symptom Rating Scale in patients with gastroesophageal reflux disease. Qual Life Res. 7, 75–83. doi: 10.1023/a:1008841022998 9481153

[B48] Ross M. C. Muzny D. M. McCormick J. B. Gibbs R. A. Fisher-Hoch S. P. Petrosino J. F. (2015). 16S gut community of the Cameron County Hispanic Cohort. Microbiome. 3, 7. doi: 10.1186/s40168-015-0072-y 25763184 PMC4355967

[B49] Sampson T. R. Mazmanian S. K. (2015). Control of brain development, function, and behavior by the microbiome. Cell Host Microbe 17, 565–576. doi: 10.1016/j.chom.2015.04.011 25974299 PMC4442490

[B50] Samson S. L. Vellanki P. Blonde L. Christofides E. A. Galindo R. J. Hirsch I. B.. (2023). American association of clinical endocrinology consensus statement: comprehensive type 2 diabetes management algorithm –2023 update. Endocrine Practice. 29, 305–340. doi: 10.1016/j.eprac.2023.02.001 37150579

[B51] Santiago-Torres M. Kratz M. Lampe J. W. Tapsoba Jde D. Breymeyer K. L. Levy L.. (2016). Metabolic responses to a traditional Mexican diet compared with a commonly consumed US diet in women of Mexican descent: a randomized crossover feeding trial. Am. J. Clin. Nutr. 103, 366–374. doi: 10.3945/ajcn.115.119016 26718418 PMC4733259

[B52] Sgritta M. Dooling S. W. Buffington S. A. Momin E. N. Francis M. B. Britton R. A.. (2019). Mechanisms underlying microbial-mediated changes in social behavior in mouse models of autism spectrum disorder. Neuron. 101, 246–59.e6. doi: 10.1016/j.neuron.2018.11.018 30522820 PMC6645363

[B53] Sherwin E. Bordenstein S. R. Quinn J. L. Dinan T. G. Cryan J. F. (2019). Microbiota and the social brain. Science. 366 (6465). doi: 10.1126/science.aar2016 31672864

[B54] Srikanth V. Sinclair A. J. Hill-Briggs F. Moran C. Biessels G. J. (2020). Type 2 diabetes and cognitive dysfunction-towards effective management of both comorbidities. Lancet Diabetes Endocrinol. 8, 535–545. doi: 10.1016/S2213-8587(20)30118-2 32445740

[B55] Stadlbauer V. Engertsberger L. Komarova I. Feldbacher N. Leber B. Pichler G.. (2020). Dysbiosis, gut barrier dysfunction and inflammation in dementia: a pilot study. BMC Geriatr. 20, 248. doi: 10.1186/s12877-020-01644-2 32690030 PMC7372911

[B56] Stolzer I. Scherer E. Suss P. Rothhammer V. Winner B. Neurath M. F.. (2023). Impact of microbiome-brain communication on neuroinflammation and neurodegeneration. Int. J. Mol. Sci. 14925. doi: 10.3390/ijms241914925 PMC10573483 37834373

[B57] Sun L. Xie C. Wang G. Wu Y. Wu Q. Wang X.. (2018). Gut microbiota and intestinal FXR mediate the clinical benefits of metformin. Nat. Med. 24, 1919–1929. doi: 10.1038/s41591-018-0222-4 30397356 PMC6479226

[B58] Svedlund J. Sjodin I. Dotevall G. (1988). GSRS–a clinical rating scale for gastrointestinal symptoms in patients with irritable bowel syndrome and peptic ulcer disease. Dig Dis. Sci. 33, 129–134. doi: 10.1007/BF01535722 3123181

[B59] Thingholm L. B. Ruhlemann M. C. Koch M. Fuqua B. Laucke G. Boehm R.. (2019). Obese individuals with and without type 2 diabetes show different gut microbial functional capacity and composition. Cell Host Microbe 26, 252–64.e10. doi: 10.1016/j.chom.2019.07.004 31399369 PMC7720933

[B60] Tremaroli V. Backhed F. (2012). Functional interactions between the gut microbiota and host metabolism. Nature. 489, 242–249. doi: 10.1038/nature11552 22972297

[B61] Turnbaugh P. J. (2017). Microbes and diet-induced obesity: fast, cheap, and out of control. Cell Host Microbe 21, 278–281. doi: 10.1016/j.chom.2017.02.021 28279330 PMC5751949

[B62] Turnbaugh P. J. Hamady M. Yatsunenko T. Cantarel B. L. Duncan A. Ley R. E.. (2009). A core gut microbiome in obese and lean twins. Nature. 457, 480–484. doi: 10.1038/nature07540 19043404 PMC2677729

[B63] Turnbaugh P. J. Ley R. E. Mahowald M. A. Magrini V. Mardis E. R. Gordon J. I. (2006). An obesity-associated gut microbiome with increased capacity for energy harvest. Nature. 444, 1027–1031. doi: 10.1038/nature05414 17183312

[B64] Umpierrez G. E. Gonzalez A. Umpierrez D. Pimentel D. (2007). Diabetes mellitus in the Hispanic/Latino population: an increasing health care challenge in the United States. Am. J. Med. Sci. 334, 274–282. doi: 10.1097/MAJ.0b013e3180a6efe3 18030184

[B65] Vallianou N. Stratigou T. Christodoulatos G. S. Dalamaga M. (2019). Understanding the role of the gut microbiome and microbial metabolites in obesity and obesity-associated metabolic disorders: current evidence and perspectives. Curr. Obes. Rep. 8, 317–332. doi: 10.1007/s13679-019-00352-2 31175629

[B66] Vintimilla R. Reyes M. Johnson L. Hall J. O'Bryant S. (2020). Cardiovascular risk factors in Mexico and the United States: a comparative cross-sectional study between the HABLE and MHAS participants. Gac Med. Mex. 156, 17–21. doi: 10.24875/GMM.19005350 32026882 PMC8785358

[B67] Vuong H. E. Yano J. M. Fung T. C. Hsiao E. Y. (2017). The microbiome and host behavior. Annu. Rev. Neurosci. 40, 21–49. doi: 10.1146/annurev-neuro-072116-031347 28301775 PMC6661159

[B68] Wright E. (2016). Using DECIPHER v2.0 to analyze big biological sequence data in R. R J. 8, 352–359. doi: 10.32614/RJ-2016-025

[B69] Yang Z. Li J. Gui X. Shi X. Bao Z. Han H.. (2020). Updated review of research on the gut microbiota and their relation to depression in animals and human beings. Mol. Psychiatry 20, (11), 2759–2772. doi: 10.1038/s41380-020-0729-1 32332994

