# Supplementary material for: Gut pathobiont enrichment observed in a population predisposed to dementia, type 2 diabetics of Mexican descent living in South Texas
Source: Front Microbiomes. 2024 Dec 3;3:1456642. doi: 10.3389/frmbi.2024.1456642 (PMC12443060; doi:10.3389/frmbi.2024.1456642)
Supplement: Supplementary file 1 [file SupplementaryFile1.docx]

**Supplemental Information**

**Gut pathobiont enrichment observed in a population predisposed to dementia, type 2 diabetics of Mexican descent living in South Texas**

Lisa M. Matz^1^, Nisarg S. Shah^2^, Laura Porterfield^3,4^, Olivia M. Stuyck^5^, Michael D. Jochum^5^, Rakez Kayed^6^, Giulio Taglialatela^6^, Randall J. Urban^2^, Shelly A. Buffington^1,7^*

**Figure S1**

^
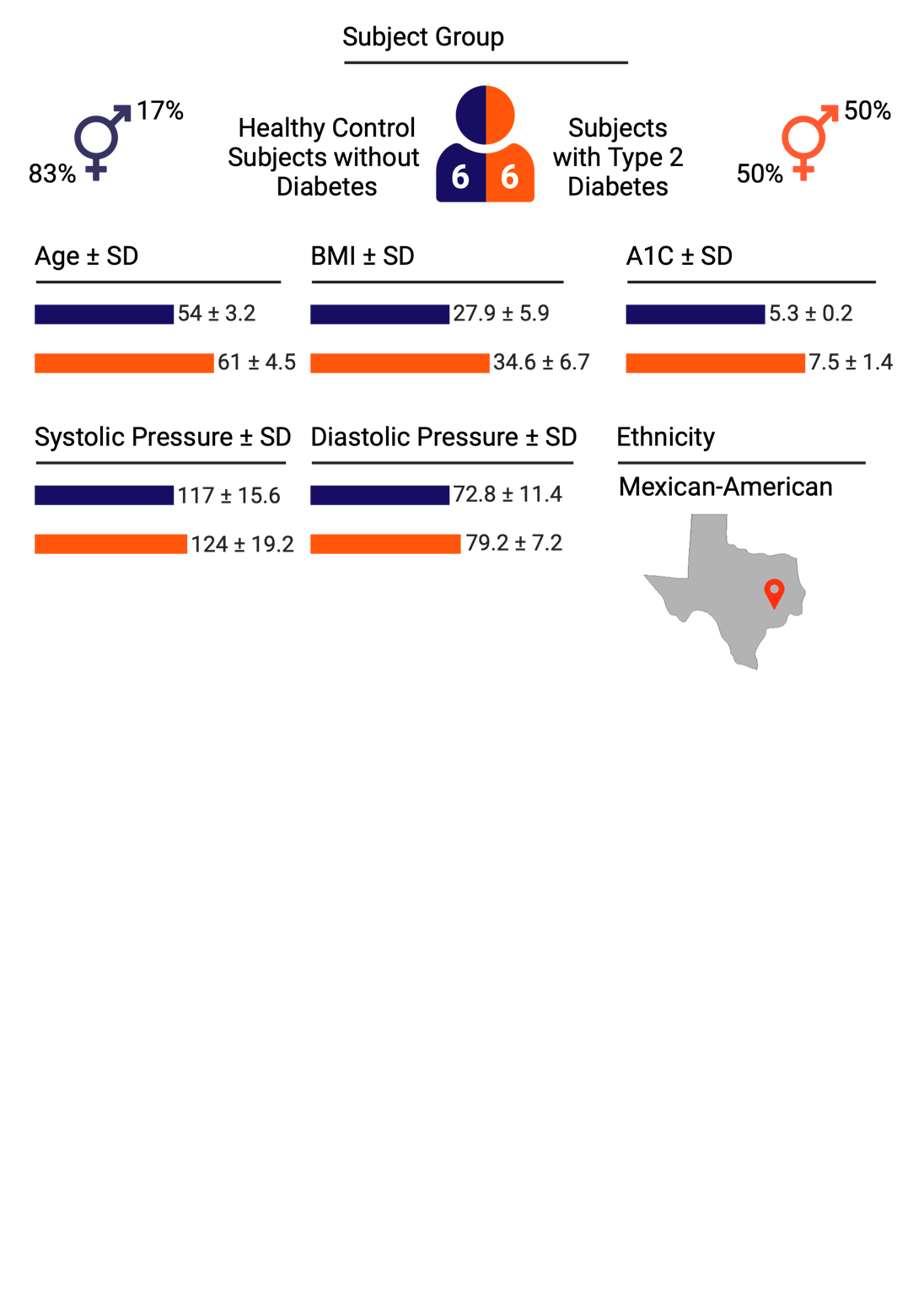
^

**Figure S1.** **Study subject demographic schematic**. Demographics of healthy controls without diabetes and subjects with type 2 diabetes as reported in Table 1. SD, standard deviation of the mean. Related to **Figure 1**, **Table 1**.

**Figure S2**

**Figure S2.** **Gastrointestinal Symptom Rating Scale scores averaged by category.** Average severity scores (y-axis) for each gastrointestinal symptom category (X-axis) did not differ significantly between HC and sT2D as determined by two-way ANOVA [Reflux: (t(45) = 2.055, *p* = 0.2084); Abdominal Pain: (t(45) = 1.028e-9, *p* = >0.9999.); Indigestion: (t(45) = 0.8478, *p* = 0.9229); Diarrhea (t(45) = 1.165, *p* = 0.7631); Constipation (t(45) = 2.364 , *p* = 0.1074)]. Related to **Figure 2**.

**Figure S3**


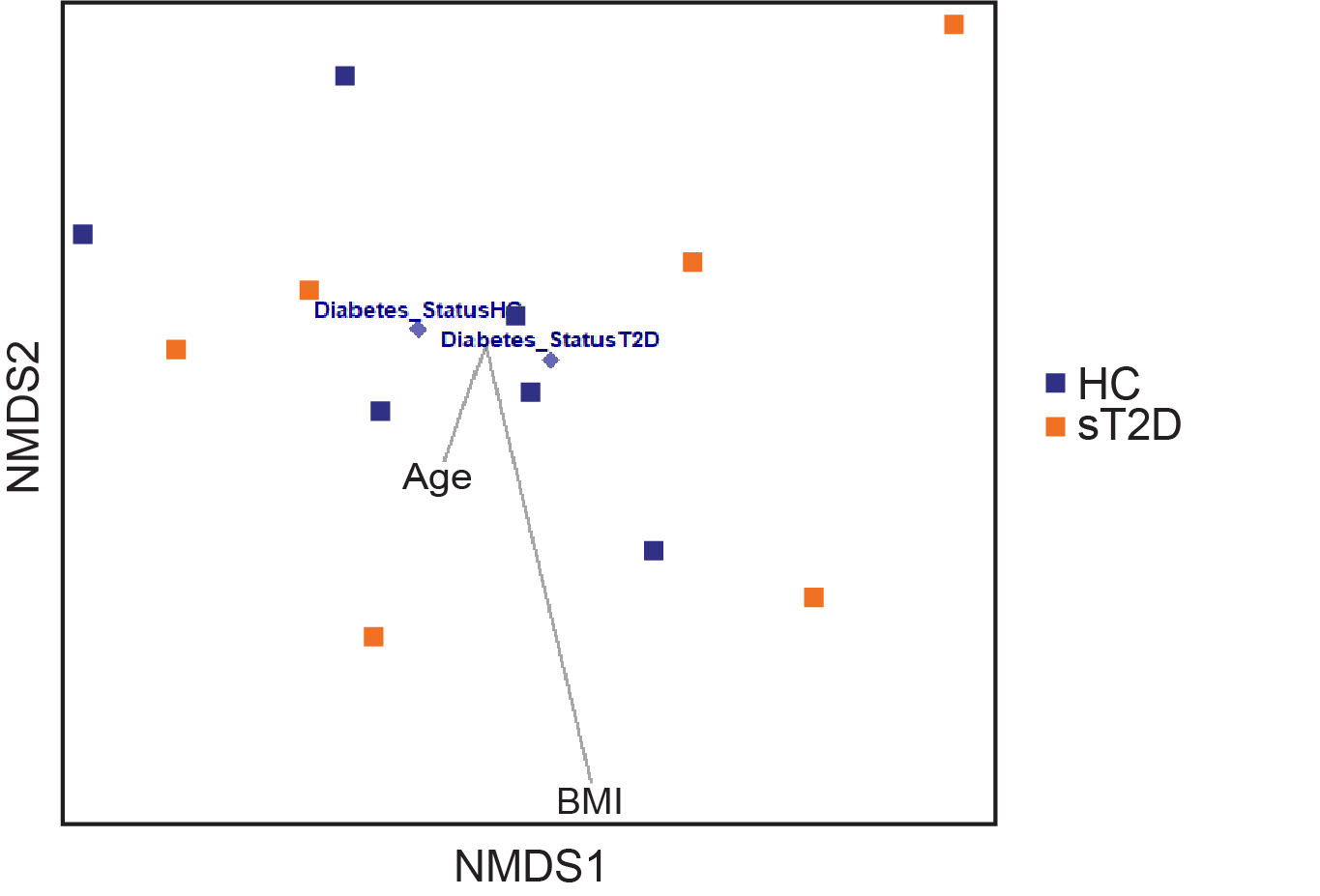


**Figure S3. Non-metric multidimensional scaling (NMDS) plot with Envfit overlay comparing HC and sT2D samples revealed that BMI, but not age, is a strong variable driving community structure.** Envfit plot incorporating vectors of BMI and Age (BMI: p = 0.007, R^2^ = 0.7062, permutations = 999; Age: p = 0.750, R^2^ = 0.0548, permutations = 999). Bray–Curtis dissimilarity was used as the distance metric for the overlay of BMI and age vectors. HC and sT2D samples shown in blue and orange, respectively. Related to **Figures 3** and **S1**.

**Table S1.**

| **Drug Category** | **# sT2D using** |
| --- | --- |
| Anti-hypertensives | 4/6 |
| Anti-diabetics | 6/6 |
| Anti-inflammatories | 3/6 |
| PPIs/H2As/Anti-histamines | 4/6 |
| Anti-depressants, anti-convulsants | 2/6 |
| Anti-lipemics | 4/6 |

**Table S1. Categories of select drugs prescribed to subjects with Type 2 Diabetes.**

**Table S2**

| **Pathway Description** | **Pathway** | **KO** | **Definition** |
| --- | --- | --- | --- |
| **Alpha-Linolenic acid metabolism** | ko00592 | K00632 | fadA, fadI; acetyl-CoA acyltransferase [EC:2.3.1.16] |
|  | ko00592 | K01058 | pldA; phospholipase A1/A2 [EC:3.1.1.32 3.1.1.4] |
| **Polycyclic aromatic hydrocarbon degradation** | ko00624 | K00448 | pcaG; protocatechuate 3,4-dioxygenase, alpha subunit [EC:1.13.11.3] |
|  | ko00624 | K00449 | pcaH; protocatechuate 3,4-dioxygenase, beta subunit [EC:1.13.11.3] |
|  | ko00624 | K00480 | E1.14.13.1; salicylate hydroxylase [EC:1.14.13.1] |
| **Bacterial invasion of epithelial cel**ls | ko05100 | K12785 | espG, virA; LEE-encoded effector EspG |
|  | ko05100 | K13085 | ipgD, sopB; phosphatidylinositol-4,5-bisphosphate 4-phosphatase [EC:3.1.3.78] |
|  | ko05100 | K13284 | sipA, ipaA; invasin A |
|  | ko05100 | K13285 | sipB, ipaB, bipB; invasin B |
|  | ko05100 | K13286 | sipC, ipaC, bipC; invasin C |
|  | ko05100 | K13287 | sipD, ipaD, bipD; invasin D |
|  | ko05100 | K13730 | inlA; internalin A |
|  | ko05100 | K13732 | fnbA; fibronectin-binding protein A |
|  | ko05100 | K13735 | yeeJ; adhesin/invasin |
|  | ko05100 | K13742 | ipgB1; protein IpgB1 |
|  | ko05100 | K13743 | ipgB2; protein IpgB2 |
| **Staphylococcus aureus infection** | ko05150 | K03367 | dltA; D-alanine--poly(phosphoribitol) ligase subunit 1 [EC:6.1.1.13] |
|  | ko05150 | K03739 | dltB; membrane protein involved in D-alanine export |
|  | ko05150 | K03740 | dltD; D-alanine transfer protein |
|  | ko05150 | K11041 | eta; exfoliative toxin A/B |
|  | ko05150 | K14188 | dltC; D-alanine--poly(phosphoribitol) ligase subunit 2 [EC:6.1.1.13] |
|  | ko05150 | K14192 | clfB; clumping factor B |
|  | ko05150 | K14201 | clfA; clumping factor A |
|  | ko05150 | K14205 | mprF, fmtC; phosphatidylglycerol lysyltransferase [EC:2.3.2.3] |
|  | ko05150 | K19079 | vraF; cationic antimicrobial peptide transport system ATP-binding protein |

**Table S2. List of KEGG orthologs (KO) contributing to significantly differentially abundant KEGG pathways (ko) in sT2D compared to HC. See also Table 2 and Supplemental File 3.**

**Questionnaires**
